# Mental contrasting as a behaviour change technique: a systematic review protocol paper of effects, mediators and moderators on health

**DOI:** 10.1186/s13643-016-0382-6

**Published:** 2016-11-25

**Authors:** Ainslea Cross, David Sheffield

**Affiliations:** 1 University of Derby Online Learning, University of Derby, Derby, UK; 2 College of Life and Natural Sciences, University of Derby, Derby, UK

**Keywords:** Mental contrasting, MCII, Implementation intentions, Self-regulation, Goal attainment, Goal pursuit

## Abstract

**Background:**

Mental contrasting is a self-regulation strategy that is required for strong goal commitment. In mental contrasting, individuals firstly imagine a desired future or health goal that contrasted with the reality proceeding the goal state, which after reflection is viewed as an obstacle (Oettingen et al. J Pers Soc Psychol 80:736–753, 2001). Mentally contrasting a positive future with reality enables individuals to translate positive attitudes and high efficacy into strong goal commitment.

**Methods:**

A systematic review of the literature is proposed to explore the efficacy of mental contrasting as a behaviour change technique (Michie et al., Ann Behav Med 46: 81-95, 2013) for health. The review also aims to identify the effects of mental contrasting on health-related behaviour, as well as identifying mediator and moderator variables.

**Discussion:**

This will be the first systematic review of mental contrasting as a health behaviour change technique. With sufficient studies, a meta-analysis will be conducted with sensitivity and sub group analyses. If meta-analysis is not appropriate, a narrative synthesis of the reviewed studies will be conducted.

**Systematic review registration:**

Review protocol registered on PROSPERO reference CRD42016034202.

**Electronic supplementary material:**

The online version of this article (doi:10.1186/s13643-016-0382-6) contains supplementary material, which is available to authorized users.

## Background

Goal setting interventions, such as mental contrasting and implementation intentions, have become increasingly popular in promoting health behaviour change [1, 3–5]. Mental contrasting involves guiding an individual to translate positive attitudes and high self-efficacy into strong goal commitment. This intervention can help guide goal pursuit behaviours, and is hypothesised to be most effective when goal attainment is challenging [3, 5]. Mental contrasting is theoretically based on the principles of fantasy realisation theory [1] (FRT). FRT posits that goal commitment is required for goal attainment, particularly when those goals are perceived as difficult to achieve [5], although this has received little empirical support. It is hypothesised that an individual’s goal commitment is founded on their high expectations of success and high incentive value held towards the goal [6], but it is possible that behaviour change could occur even when the individual’s expectation of success is not high (particularly so in the case of the difficulty of achieving long-term weight loss), but the incentive value is high.

Evidence of the effectiveness of mental contrasting as a behaviour change technique for improving motivation and performance is growing [3–11]. Studies have assessed goal pursuit using various indicators including cognitive (e.g. making plans), affective (e.g. feelings of anticipated disappointment in case of failure), motivational (e.g. feelings of determination), physiological (e.g. energization assessed by cardiovascular measures), and behavioural (e.g. number of initiated actions) measures. Effects have been observed regardless of whether these indicators were self-reported or observed, whether they were assessed immediately after the mental exercise or weeks later, and whether mental contrasting was experimentally induced or self-generated [12–15].

There is emerging psychophysiological evidence that mental contrasting operates by energising individuals thus providing the resources for behaviour change. This energization has been studied through changes in systolic blood pressure [15, 16]; when perceived expectation of chances of success were high, mental contrasting increased systolic blood pressure (SBP), whereas when they were low, mental contrasting decreased SBP. Moreover, changes in energization have been suggested as a mediator for the relationship between expectations and goal pursuit [17]. It is hypothesised that mentally indulging on a desired goal without performing mental contrasting uses too much energy and is then detrimental to achieving the individual’s desired behaviour change. Mental contrasting is hypothesised to mobilise sufficient energy resources to cope with obstacles in order to attain goals and facilitate goal pursuit. Importantly, mental contrasting conserves energy resources and reduces the probability of depletion and exhaustion thus permitting behavioural flexibility in responding to urgent environmental demands and accomplishing behaviour change goals [16].

In order to address this challenge and to understand the most effective active ingredients of interventions, the behaviour change taxonomy [2] aims to create a shared language. Thus, the BCT taxonomy facilitates the design and evaluation of effective interventions and is used to synthesise evidence about the effectiveness of mechanisms underlying behaviour change. Version one of the taxonomy specified 93 hierarchically clustered behaviour change techniques (BCTs). Mental contrasting most strongly relates to the goals and planning hierarchical cluster, which specifically includes the BCTs 1.1. Goal Setting (behaviour), 1.2 Problem Solving and 1.3 Goal Setting (outcome). This accords with the theory of planned behaviour study findings [18] that goal setting and forming intentions to change behaviour alone do not necessarily result in behaviour change. Mental contrasting may bridge this intention-behaviour gap (TPB) by requiring an individual to imagine a desired future or health goal, and to then contrast their goal with the reality preceding the goal state. This reflection on goals and intention requires the individual to identify potential obstacles and to form plans that increase the chances of the behaviour being performed [1].

In summary, mental contrasting shows promise as a BCT for improving health-related behaviours due to its ability to raise goal commitment and performance when perceived expectations of success are high, rather than allowing the individual to indulge and dwell [1, 7–9, 11]. Accordingly, mental contrasting is proposed as a self-regulation strategy that translates expectancy into behaviour.

### Mental contrasting and implementation intentions (MCII)

More recently, research has begun to focus on the combination of mental contrasting and implementation intentions (MCII) for improving health. Implementation intentions are simple action plans that follow an if–then format by specifying when, where and how a goal intention should be implemented into action [19]. After mental contrasting, the formation of implementation intention may help to translate desires into behaviour. Forming an if–then plan creates a perceptual readiness to recognise the critical cue and link it to a specific goal-directed behaviour. When the cue is then encountered, the goal-directed behaviour is automatically enacted without the need for conscious intent. Implementation intentions have been shown to facilitate goal attainment in laboratory experiments using standardised performance tasks, as well as in field studies using complex behavioural tasks. They have been shown to be effective in various domains such as achievement, interpersonal relations and health, as revealed in a meta-analysis of 94 studies [20].

## Objectives

This is the first review to systematically identify and evaluate the effectiveness mental contrasting as a behaviour change technique for improving physical health-related behaviours and outcomes. The aims of this systematic review are to answer the following questions:

- How effective is mental contrasting as a BCT for improving health-related behaviours in adult populations compared with control or active control groups?
- What are the mediators (e.g. energization) and moderators (e.g. age, gender, type of behaviour) of mental contrasting on health-related behaviours?
- Does the addition of implementation intentions to mental contrasting (MCII) lead to improvements in the effectiveness of mental contrasting?

### Review methods

This protocol has been reported using the Preferred Reporting Items for Systematic Reviews and Meta-Analyses Protocols (PRISMA-P) guidelines [21], included as Additional file 1.

## Eligibility criteria

### Study characteristics

This review will include published, unpublished and in-progress randomised control trial intervention studies from 1995 to present (2016). Randomised control trials will be included in the review as they provide the gold standard of evidence and are most suited to evaluating the effectiveness of mental contrasting interventions.

### Participants

This review will include only studies with adult participants (18 or older) with no upper age limit.

### Intervention

Studies will be included if they describe a randomised control trial evaluating a mental contrasting intervention, or use mental contrasting for physical health behaviour (e.g. exercise, smoking cessation, diet) as one of its primary aims. Intervention approaches must include at least one BCT from the BCT taxonomy v1 [2], hierarchical cluster 1: ‘Goals and Planning’, but do not need to have a specified theoretical basis. Where BCTs are noted to come from hierarchical clusters other than ‘Goals and Planning’, these will be recorded and reported. Interventions can be delivered in a variety of settings (e.g. university, community centres, hospital, clinic, private residence) or modes of delivery (e.g., face-to-face, online, text message, phone call). Studies will be excluded if they do not report using mental contrasting and/or they report outcomes other than health-related behaviour, for example, education or time management.

Papers will be entered into the review where they are available in full text, report primary quantitative data from randomised controlled trial designs, and are either published within peer-reviewed sources, or, for registered trials only, unpublished but completed and with results obtainable from the authors. Studies from 1995 onwards will be included as the theory, and approach for mental contrasting was first described at this point. Professional translation will be sought for non-English language papers. Where results from the same trial are reported across multiple sources, information from these sources will be pooled and treated as a single trial for the purpose of analysis.

### Comparator or control

This review will include randomised control trials studies that compare a mental contrasting intervention that contains at least one BCT to either passive control groups (e.g. usual care, waiting list control, no treatment), active control groups or passive control groups.

### Outcome measures

Primary outcomes: Health-related behaviour outcomes (e.g. increasing physical activity, healthy eating or smoking cessation), self-report, observation, behavioural and physiological outcomes. Objective outcomes will be prioritised in terms of the following hierarchy of behavioural outcomes:

1. Objectively measured behaviour, e.g. number of steps as measured by a pedometer
2. Observed behaviour, i.e. a sampled number of observations, such as a health care professional observing and reporting a patient’s step count
3. Physiological changes related to behaviour, e.g. reported changes in heart rate following physical activity
4. Ecological momentary self-report of behaviour, e.g. diaries or real time monitoring of steps
5. Retrospective self-report, e.g. questionnaires of health behaviour requiring retrospective recall

#### Secondary outcomes

For the studies that meet the principal inclusion criteria, the following outcomes will also be assessed if available: type of effects (i.e. classification), dropout (i.e. data on participants who set a mental contrasting behavioural goal but are lost to follow-up), mediator and moderator variables.

### Information sources

This review will include comprehensive searches on the following electronic databases: Databases to be searched include: Scopus (1960 to present), PsycINFO (1966 to present), CINAHL (1982 to present) and Web of Science Core Collection (1970 to present). In addition to the electronic database searches, relevant conference proceedings from 1995 onwards will be searched to identify unpublished studies.

We will also search for published systematic reviews of behaviour change in the Database of Abstracts of Reviews of Effects (DARE) and the Cochrane Database of Systematic Reviews (CDSR). Ongoing research will be identified through the National Institute of Health Research (NIHR) portfolio for recently completed or ongoing studies, ongoing trial registers (http://www.isrctn.com/ and www.clinicaltrials.gov) and the System for Information on Grey Literature (SIGLE) will be searched for further grey literature. Furthermore, we will hand search the bibliographies of all included studies and request from experts in the field any relevant information on unpublished and ongoing research and key-related journals from our scoping search including the British Journal of Health Psychology, Journal of Health Psychology and Psychology and Health.

### Search strategy

Searches will include a combination of terms from medical subject headings (MeSH) and keywords in the title, abstract and text for the intervention, comparator and outcomes using the terms in Table 1.

**Table 1:** Search terms to be used

Concept	Search terms
Intervention	mental* contrast*, mental* contrast* and self-regulation, Mental* contrast* Imp* intention*, MCII and self-regulation, mental* contrast*and goal*, mental* contrast*and goal setting, mental* contrast* and goal pursuit, mental* contrast* and goal projection, mental* contrast* and goal attainment, mental* contrast* and Expectancy, mental* contrast* and fantasy and future and behaviour change, mental* contrast* and fantasy and future and behavior change
Study type	Clinical trial [pt], randomly [ab], randomized [ab], trial [ti], clinical trials
Outcomes	mental* contrast* and health, mental contrasting (exp) and health, MCII and health, mental* contrast* and behaviour*, mental* contrast* and behavior*, mental contrasting (exp) and behaviour, mental contrasting (exp) and behavior*, MCII and behaviour*, MCII and behavior*

## Data management

The results from all literature searches will be imported into the Endnote reference management software, with duplicates removed by the main reviewer.

## Selection process

One reviewer will screen all retrieved records by title and abstract for all inclusion and exclusion criteria. A second reviewer will also screen a random 20% of the total titles and abstracts. Any disagreements at this stage will be included for further assessment. Following initial screening, full-text versions of all potentially relevant studies will be retrieved and reviewed independently for suitability (using the inclusion and exclusion criteria to guide study selection choice) by two reviewers. Study authors will be contacted where necessary if relevant information on eligibility is missing. Reasons for inclusion and exclusion will be recorded, and a group discussion will resolve any discrepancies following a blind review by a third researcher.

## Data extraction

Two independent reviewers (AC and DS) will complete a data extraction (using PRISMA and QUOROM guidelines) form for each selected study and will be assessed for bias against the Cochrane tool for bias. The reviewers will independently extract data from included studies into Excel using the data extraction form. Any disagreements will be resolved by discussion with a third researcher and/or by seeking further clarification from study authors.

### Data items

Two reviewers will code and extract data independently using the following categories based on the template for intervention description and replication (TIDIER) checklist of information to include when describing and reporting interventions [22]

- General: date of data extraction, author(s), article title, type of publication, country of origin and source of funding
- Study characteristics: aims/objectives of the study, description of comparator/control group features, inclusion and exclusion criteria, details of pre-protocol registration, source of funding, recruitment and sampling methods (including unit of randomisation and blinding), fidelity measures and unit of allocation
- Participants: population type and setting, inclusion and exclusion criteria, number of participants, and baseline characteristics (e.g. age, gender, weight status, ethnicity, socioeconomic status and health/risk factors)
- Features of interventions: description of the intervention(s), intervention setting, control condition(s), coding of BCTs based on the BCT taxonomy v1 [2], guidelines for reporting of interventions, and theoretical basis, mediator and moderator variables
- Measurement description: unit of measurement, type of measurement used (objective/subjective), additional outcomes measured, follow-up duration, and frequency

Where possible, we will include results that have used intention-to-treat analysis, and if effect sizes cannot be calculated, further information will be sought from study authors. The study authors will be contacted to supply missing information where necessary. If information is not forthcoming from this process, the most conservative estimates will be made using available data, i.e., at the lower 95% confidence interval (CI).

### Risk of bias in individual studies

Two reviewers will independently assess the methodological quality of each of the studies using the Cochrane tool for assessing risk of bias. This tool evaluates the quality of allocation sequence generation and concealment; blinding of participants, intervention providers and outcome assessors; completeness of data; the extent to which outcomes are selectively reported; and any other potential sources of bias. Each study will be rated for bias as either: ‘low risk for bias’, ‘unclear risk for bias’ and ‘high risk for bias’. Information on quality for each study will be accompanied by a description of the assessment and decision-making process. Further, AMSTAR criteria will be followed to evaluate and reduce the potential for bias in the review [23].

### Data synthesis

Differences in effectiveness will be analysed according to outcomes and the number and type of BCTs used in each review study. Appropriate statistical techniques will be used for each type of continuous (weighted mean differences if outcomes are consistent or standard mean difference if different outcomes are used, with 95% CI) and dichotomous variables (risk ratios, with 95% CI). This review will also include a meta-analysis (if there is sufficient homogeneity of outcomes) to calculate pooled effect sizes across studies, using a random-effect model. Heterogeneity will be investigated in each study using χ^2^ (significance level: 0.1) and Higgins I^2^ statistics, with high levels (as specified by guidance in the Cochrane Handbook for Systematic Reviews of Interventions [24]) being considered suitable for subgroup analysis to determine the source of the heterogeneity.

If a meta-analysis is not possible, a narrative synthesis of all relevant studies will be conducted, including tables of study characteristics, participant and intervention details, settings and outcomes.

### Subgroups and sensitivity analysis

Analysis by subgroups will include (if possible or appropriate) the following: mode of delivery (e.g. face-to-face or internet-delivered); BCTs; theoretical basis; and targeting single versus multiple health outcomes/behaviours, where possible subgroup analysis for categorical predictors will be undertaken, along with meta-regression for metric predictors. Sensitivity analysis will be carried out to determine the effects of studies with a high risk of bias on the overall results with and without these studies. Mediators are likely to include (but not limited to) energisation, and moderator variables may be age, gender or type of health behaviour.

### Meta-bias

This review will assess study protocols for outcome reporting bias by judging whether authors have selectively reported outcomes using the Cochrane tool for assessing risk of bias. Reporting bias will be analysed using statistics and forest plots.

### Confidence in cumulative evidence

The quality of evidence for primary outcomes from each of the studies included in the review will be assessed using the Grading of Recommendations Assessment, Development and Evaluation (GRADE) guidelines [25], which include the following domains: design, study limitations, consistency, directness, precision and publication bias. Each study and its relative outcomes will be assessed using the GRADE guidelines. The quality of each review study will be judged as high (for studies in which we have a high level of confidence that the true effect lies close to that of the estimate of the effect), moderate (where we can be moderately confident in the effect estimate: the true effect is likely to be close to the estimate of the effect, but there is a possibility that it is substantially different), low (where our confidence in the effect estimate is limited because the true effect may be substantially different from the estimate of the effect), or very low (we have very little confidence in the effect estimate: the true effect is likely to be substantially different from the estimate of effect).

## Discussion

This systematic review will provide a detailed overview of the existing evidence base for mental contrasting interventions that have been designed to improve health outcomes and health-related behaviour. It will also consider the effectiveness of the self-regulation strategy of mental contrasting as a behaviour change technique. Furthermore, examining the use of behaviour change techniques within mental contrasting interventions may provide clarity on the effective components of mental contrasting interventions. This will afford recommendations to be made about whether mental contrasting is effective as a stand-alone intervention, or whether the addition of other behaviour change techniques (such as those in implementation intentions) result to more improvement in health outcomes and health behaviours. Examining mediators may enable inferences about probable mechanisms of action to be made. Possible moderators may allow determination of those who are most likely to benefit from mental contrasting.

Our interpretation of the review studies will take into account limitations in review studies that have identified by the authors, along with any limitations identified by our review methodology. In conclusion, this is the first review to systematically identify and evaluate the effectiveness mental contrasting as a technique to change health behaviour.

## Additional file

Additional file 1: Mental contrasting as a behaviour change technique: a systematic review protocol paper of effects, mediators and moderators on health. Preferred Reporting Items for Systematic review and Meta-Analysis Protocols (PRISMA-P) 2015 checklist: recommended items to address in a systematic review protocol. (DOC 115 kb)

## Abbreviations

AMSTAR: A Measurement Tool to Assess Systematic Reviews
BCT: Behaviour change technique
GRADE: Grading of Recommendations Assessment, Development and Evaluation
MCII: Mental contrasting and implementation intentions
PRISMA: Preferred reporting items for systematic reviews and meta-analyses
QUOROM: Quality of reporting of meta-analyses
TIDIER: Template for intervention description and replication
